# Large-scale assessment of human navigation ability across the lifespan

**DOI:** 10.1038/s41598-020-60302-0

**Published:** 2020-02-24

**Authors:** Ineke J. M. van der Ham, Michiel H. G. Claessen, Andrea W. M. Evers, Milan N. A. van der Kuil

**Affiliations:** Department of Medical, Health and Neuropsychology, Leiden University, Leiden, the Netherlands

**Keywords:** Cognitive ageing, Cognitive neuroscience, Neurology

## Abstract

Navigation ability is particularly sensitive to aging. Evidence of aging patterns is largely restricted to comparing young adults and elderly and limited in the variety of navigation tasks used. Therefore, we designed a novel task battery to assess navigation ability in a very large, representative sample (N = 11,887, 8–100 years). The main aim was to measure navigation ability across the lifespan in a brief, yet comprehensive manner. Tasks included landmark knowledge, egocentric and allocentric location knowledge, and path knowledge for a route and survey perspective. Additionally, factors that potentially contribute to navigation ability were considered; gender, spatial experience and spatial anxiety. Increase in performance with age in children was found for allocentric location knowledge and for route-based path knowledge. Age related decline was found for all five tasks, each with clearly discernible aging patterns, substantiated the claim that each task distinctively contributes to the assessment of navigation ability. This study provides an in depth examination of navigation ability across dissociable functional domains and describes cognitive changes across the lifespan. The outcome supports the use of this task battery for brief assessment of navigation for experimental and clinical purposes.

## Introduction

Whether it is a matter of finding our way to the kitchen in the morning, or travelling to a foreign country for the first time, we are constantly making use of our navigation ability. We use a range of different cognitive processes when we navigate. For instance, we identify landmarks, keep track of our goal location, and calculate the shortest route to that goal location. Numerous studies have supported such dissociable cognitive aspects with distinct behavioral and neurological performance patterns^1–4^. In line with the variety of processes involved, navigation ability is subject to large individual differences. Age plays a prominent role in these differences, as first evidenced by studies using maze tasks in rodents (e.g.^5^). Similar aging effects have been shown in humans. Spatial navigation has been assessed in young and older adults, showing clear indications of age related decline for some aspects of navigation ability, dependent on their proposed neural correlates^6–8^. Such decline in navigation ability in turn leads to more incidents of getting lost, which inhibits optimal participation in society for an increasing part of the population. The last few years have seen a steep increase of interest in how navigation ability is affected by age. The pivotal role of the hippocampal areas in navigation makes this cognitive ability one of the first to decline with age, as these brain structures in particular substantially change during the aging process^9,10^. Moreover, in pathological aging, getting lost is one of the earliest and most prominent signs of the disease process (e.g.^6^).

As the existing evidence of aging patterns in navigation is restricted both in terms of age range and the variety of navigation tasks used, the main aim of the current study was to create a detailed, large-scale depiction of navigation ability across the lifespan. Studies to date have typically reported differences between very young and very old individuals, and have often administered a small selection of tasks, representative only partly of what can be considered navigation ability. Therefore, the current study was designed to include participants across the lifespan, to address navigation ability in a very large sample of participants with clear measurements of navigation ability at a behavioural level. To create a more comprehensive measure of navigation ability, we considered current theoretical frameworks in the literature and created a task battery consisting of a range of different tasks to reflect the cognitive complexity of navigation.

To motivate a meaningful selection of tasks, the literature does not provide a straightforward answer yet. Existing lines of navigation research consider navigation ability a cognitive function consisting of multiple components. Therefore, multiple tasks should be included, to reflect this range of functionally dissociable components. One of the most commonly cited overviews of navigation is that of Wolbers and Hegarty^1^, who organize most processes involved in navigation according to the processes’ link to the perception, processing, and representation of relevant information. Although it is highly informative and useful to list the processes of interest in these three categories, this approach does not focus on the functionality of these processes. For example, landmark identity memory is present in all three phases, but is probably a unitary function; as is illustrated by landmark agnosia, for instance^11,12^. Therefore, it is more appropriate to consider landmark identity memory as one of the components of navigation ability, rather than as a feature that is present in multiple phases, when using this functional approach to the cognitive ability of navigation.

In line with this reasoning, here we focused on three main, functionally dissociable domains of navigation; landmarks, locations, and paths. This subdivision is the result of extensive literature review, taking into account theoretical, experimental, and clinical papers, as well as recent empirical evidence^13–15^. In^16^, we have systematically reviewed all methodologically sound case studies concerning navigation impairment. All of the navigation impairment cases can be assigned to one or more of these domains, which emerge as functionally dissociable. Moreover, this approach integrates existing terminology in a meaningful way. The subdivision into landmark, route and survey knowledge was first introduced by Siegel and White^17^, who proposed that these types of knowledge were sequentially accumulated and increased in difficulty. However, empirical evidence has been insufficient to demonstrate the sequential properties proposed in the Siegel and White model (e.g.^18^) and their model does not incorporate the use of egocentric and allocentric perspectives^14,15^. Yet, many have studied egocentric and allocentric perspective taking as a key element of navigation ability, stressing its importance^19,20^. To be inclusive of such theoretical contributions, here, we specifically address route versus survey knowledge, as well as egocentric and allocentric perspective taking.

*Landmarks*, or the ‘What?’ aspect of navigation, concern the ability to identify stable elements in an environment in a spatially meaningful way (e.g.^21^). *Locations* concern the ‘Where?’ of navigation ability and reflect the processing of locations in an environment. Location knowledge fits well with the commonly drawn distinction between egocentric and allocentric processing (e.g.^22^). A particular location can be coded either as being ‘to my left’ (egocentric), or ‘north of city hall’ (allocentric). Egocentric processing concerns an observer-based or first person perspective, which is mainly linked to parietal cortex activation. Consequently, indications of location in an egocentric way concern directional information from the observer’s current or past position in an environment. In contrast, allocentric processing makes use of an environment-based perspective, disregarding the observer’s current position, clearly linked to hippocampal activation. Therefore, allocentric location knowledge can be expressed as indications of location in relation to other elements in the environment, irrespective of the observer’s previous interaction with the environment. The third category, *paths*, is the most complex and concerns the question of ‘How to get there?’. It involves the spatial context of a given landmark, which reflects how the landmark location relates to one or multiple other elements in an environment. The frequently made distinction between route versus survey knowledge (e.g.^23^) is applicable here: path knowledge may concern either a specific route one can take to reach a certain location (route knowledge), or allow a representation of a spatial configuration from a bird’s eye perspective (survey knowledge). In short, to obtain meaningful objective measures of navigation performance, we made use of a task battery that includes measures for each of these three navigation domains.

### Aging patterns

With regard to age, differential patterns were hypothesised for each of these domains, which further substantiates the dissociation between them. Landmark knowledge is often not included in existing aging studies and shows mixed effects. Given its connection with the parahippocampal place area and striatum^24,25^, a shallow decline was expected, with relatively early onset. Where location knowledge is concerned, both egocentric and allocentric, the literature so far shows a relative consensus. Aging effects are observed in allocentric tasks, due to the links to the hippocampus, which is especially sensitive to healthy aging^26^. Egocentric aspects of navigation ability remain relatively intact throughout aging due to this ability’s connection to the parietal cortex^8,27^. When it comes to path knowledge, a pattern of early decline was hypothesized for route knowledge of paths, given the role of the medial temporal lobe including the hippocampus in route retrieval^28^. For survey knowledge of paths, neural correlates also include the inferior temporal cortex and posterior superior parietal cortex, potentially moderating the aging effect related to the temporal cortex^29^.

### Developmental patterns

Not only the impact of aging has been a topic of interest with regard to navigation ability. Also, cognitive development is highly relevant within this context. It allows to determine when (young) adult levels of performance are reached and can be linked to neuroanatomical measures, similar to performance at an older age. Although many of the developmental studies on spatial cognition concern very young participants, due to the nature of the current experimental set-up, we chose to include participants from age 8 and upwards. This allowed us to use the same task for all participants, and assured sufficient reading abilities and understanding of the task instructions for all included ages. Given the distinct neurological and functional properties of the three domains of navigation, we hypothesize separate developmental patterns for each navigation domain. This would further substantiate the distinctions between these domains. For landmark knowledge, it has been found that younger children, at the age of 8 rely more heavily on landmarks compared to 12 and 24 year olds, as they are hindered more by removal of landmarks in a navigational task^30^. So younger children may focus more on the landmarks in an environment than on more complex spatial features. In contrast, location knowledge may reach adult performance at a later age, in particular allocentric location knowledge. Adolescents at the age of 14 are still inferior in translating information allocentrically when they are asked to label a map^31^. For path knowledge, 8 year old children still lack integrated knowledge about the configuration of space, whereas 12 year olds have achieved a relatively good grasp of integrated route information. Survey level knowledge has been found to reach an optimal level later, at adult age^30^. In addition, Pine and colleagues^31^ also report an adult level of route knowledge is achieved at age 14, as goal locations can be found equally well at this age.

### Gender, spatial experience, and spatial anxiety

Apart from age, other variables have been found to impact an individual’s navigation ability, of which gender may be the most prominent. A male advantage is frequently found, but may be linked to visuospatial working memory and only occur when the load on visuospatial working memory is high (see^32^). Others argue that effects of gender are mainly linked to a different use of strategies, as reflected by dissociable neural correlates found in males and females^33^. A commonly found difference lies not with the level of performance itself, but with the type of information that is considered by males and females. Females tend to favour landmark information, whereas males also consider geometric information (e.g.^34^). In terms of the three domains of navigation, this would mean that there is no general male advantage in navigation performance, but that males are expected to outperform females when more geometric processing is required. Both egocentric and allocentric location knowledge and survey path knowledge, require a substantial level of geometric processing and are therefore expected to show a male advantage, whereas landmark knowledge and route path knowledge rely heavily on landmark processing and are therefore expected to lead to equal performance of males and females.

In search for an explanation for the large variety in navigation performance between individuals, many researchers have considered additional predictors. Spatial experience has been shown to affect navigation performance, as evidence for instance by neurological changes for certain professions which rely heavily on navigation skills, such as taxi drivers^35,36^. Given that the size of our sample was so large and participants were recruited throughout the Netherlands and Belgium, we could also consider living area as a potential factor. Although very little research has been done on this factor, it can be argued that living area can be considered a marker of the type of spatial experience someone has. Living in a rural or urban area could differentially affect how spatial information supporting navigation is processed. Especially in the Netherlands, the flat landscapes in rural areas create high visibility of the spatial layout of the environment and distant landmarks, such as church towers or farm buildings, favouring allocentric location and survey path knowledge. In contrast, highly crowded urban areas may stimulate the use of route-based processing of paths and (proximal) landmarks. In addition, outside of the cognitive domain, also other factors, such as anxiety may also play a role. Spatial anxiety has been shown to correlate strongly with navigation performance and higher levels of spatial anxiety may even cause lower performance^37^. Therefore, we also included a measure reflecting the level of spatial anxiety for each participant to explore this further.

In short, we have assessed navigation ability and several demographic variables in a very large sample of Dutch and Belgian individuals. We used a novel task battery including measures of landmark, location and path knowledge to measure navigation ability in a functionally comprehensive way. Moreover, we have used this task battery to asses navigation ability across the lifespan and the impact of gender, spatial experience, and spatial anxiety on navigation performance. Age related declined was hypothesized for landmark knowledge, allocentric location knowledge and path knowledge, whereas egocentric location knowledge was expected to remain relatively stable with age. Males were expected to outperform females only when geometric cues are particularly necessary; for egocentric and allocentric location knowledge and survey path knowledge. Spatial experience was expected to improve navigation performance, whereas spatial anxiety is thought to result in lower performance. In addition, effects of living environment were explored.

To reach such a large population of participants, we set up an online experiment in collaboration with a national science event, organised by the Dutch government. This lead to a sample of 11,887 participants, ranging in age between 8 and 100. Due to this wide age range, we will first focus on the developmental patterns in performance, by comparing the 8–17 year olds to each other and a sample of young adults. Next, we will present the data for all adult participants (age range 18–100). Both datasets originate from the same experimental design.

## Methods

### Participants

A total of 1022 children in the age 8–17 years, and a group of 1259 young adults (18–24 years old) completed the experiment. The descriptive statistics for each age group are provided in Table 1. The adult sample consisted of a total of 10,865 individuals with ages between 18 and 100. The descriptive statistics for each age group are provided in Table 2. The study was approved by the local ethical committee at Leiden University, and in accordance with the declaration of Helsinki (2013) each participant provided informed consent prior to the experiment. For participants under the age of 16, both the participant and the participant’s parents or legal guardians were required to give informed consent in order to take part in the experiment. Individuals with neurological or psychiatric conditions were explicitly asked to abstain from participation. The general public was invited to participate through a variety of national and local media, organised by The Weekend of Science, a Dutch annual event organized by the Secretary of Education, Science and Culture, to promote science to the general public. Data collection ran from October 2017-November 2018. The experiment was introduced as a serious and formal experiment, open to all healthy individuals, aged 8 and up.

**Table 1:** Descriptive statistics of participants (age 8–17).

Age group	N	N Female	% Female	% Urban	% Navigation experience	Spatial anxiety (1–7)
8	73	32	43.8	84.9	45.2	4.33 (1.72)
9	86	36	41.9	89.5	59.3	4.26 (1.99)
10	99	48	48.5	87.9	76.8	3.45 (1.87)
11	84	38	45.2	91.7	89.3	3.39 (1.96)
12	92	33	35.9	79.4	—	3.24 (1.91)
13	56	31	55.4	73.2	—	3.04 (2.11)
14	113	57	50.4	67.3	—	3.11 (1.61)
15	64	42	65.6	81.3	—	2.83 (1.66)
16	182	106	58.2	84.6	—	2.91 (1.69)
17	173	107	61.8	76.9	—	2.72 (1.61)
18–24	1255	868 (4 nonbinary)	69.2	79.8	—	2.85 (1.76)

**Table 2:** Descriptive statistics of participants (age 18–100).

Age group	N	Mean age (SD)	% Female	% Male	% Nonbinary	Education level	% Urban	Spatial experience	Spatial anxiety
18–29	1891	23.1 (3.3)	67.0	32.6	0.42	6.36 (0.71)	80.0	2.49 (0.64)	2.72 (1.72)
30–39	1015	34.5 (2.8)	60.3	39.6	0.1	6.19 (0.85)	74.5	2.43 (0.65)	2.42 (1.68)
40–49	1338	44.9 (2.9)	64.7	35.4	0	6.10 (0.81)	72.4	2.33 (0.60)	2.62 (1.80)
50–59	2312	54.9 (2.8)	68.5	31.5	0.04	5.92 (0.81)	65.6	2.27 (0.59)	2.88 (1.93)
60–69	2801	64.4 (2.9)	63.6	36.4	0.04	5.84 (0.83)	64.9	2.17 (0.55)	3.06 (1.98)
70–79	1318	73.1 (2.9)	51.9	48.0	0.08	5.84 (0.90)	64.6	2.05 (0.49)	3.02 (2.02)
80–100	190	83.3 (3.6)	43.7	55.8	0.53	5.77 (1.05)	66.3	1.9 (0.54)	3.12 (2.06)

### Materials

The experiment was made available through a web-based environment (www.navigerenkunjeleren.nl), set up with Qualtrics software. The experiment could be performed on a regular computer or a mobile device. The content for both options was identical, only the format of the questionnaire was adjusted to screen size, with a vertically or horizontally positioned Likert scale for mobile devices and computer screens, respectively. The experiment consisted of a questionnaire part, a navigation experiment, and an additional, optional questionnaire, which will not be discussed here. Total duration was around 10 minutes.

The *questionnaire* consisted of demographic questions concerning gender and age. For living area participants were asked whether they lived in a rural or urban area. Examples were provided to ensure clear understanding of the question; e.g. Randstad (large urban area in the Netherlands including both Amsterdam and Rotterdam) or Terschelling (small, rural island). For children, spatial experience was measured by asking how children travelled to school (walking or biking by themselves, being brought by an adult, taking public transportation). This allowed for a distinction between more and less autonomous navigation experience. Due to its relevance especially to younger children, this question was only asked 8–11 year olds. For all adult participants spatial experience was assessed with the question ‘How often do you travel to places you have not visited before’, with response options; never, several times a year, several times a month, weekly or more. Also, education level was added as a question for adults, with all the levels available in the Dutch school system as response options, which were later recoded to the description of Verhage^38^, resulting in scores ranging from 1 (lowest) to 7 (highest). Spatial anxiety was assessed by using one of the items of the Wayfinding Questionnaire, most predictive of the spatial anxiety subscale^39,40^; ‘I am afraid to get lost in a city I do not know’, with response options 1 (totally disagree) to 7 (totally agree).

**Figure 1:**
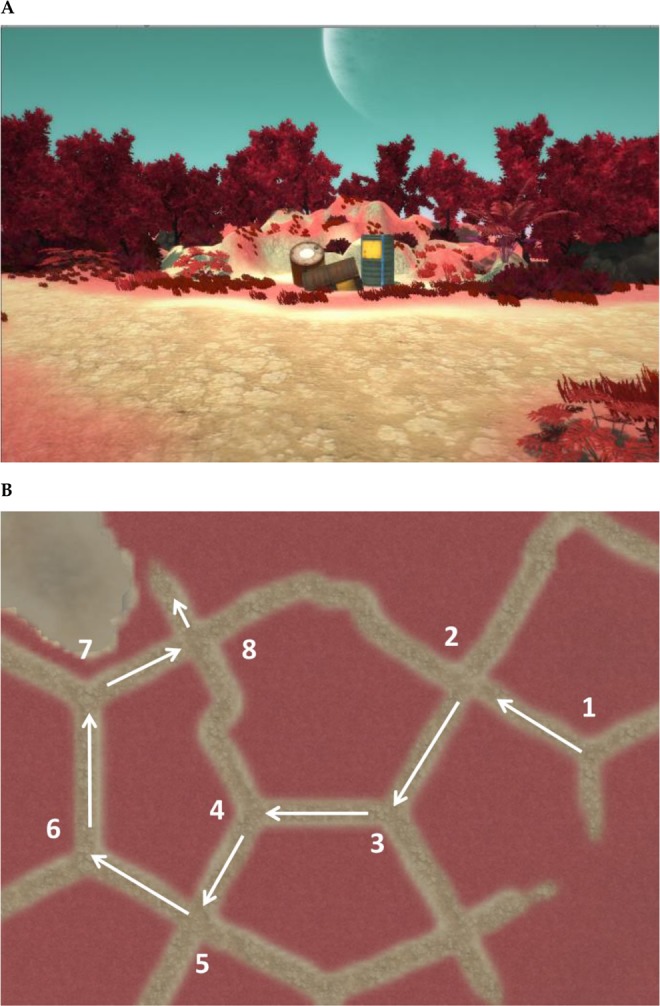
(**A**) A screenshot taken from the route depicted in the experiment. (**B**) The layout of the route, in which each number represents the position of a landmark.

For the *navigation experiment*, a short video was shown (69 s), of a route through a forest-like environment with muted colours. The route lead past eight distinguishable landmarks, with salient colours (oil drums, a shield, a crate, a boat, a car, a shipping container, a gemstone, and a buoy) placed at separate intersections. At the endpoint of the route, a spaceship was placed. The layout of the route is shown in Fig. 1. The narrative used was that the participant had landed on an unknown planet and through the video would find their way to the space ship that could take them back home. The instructions were to pay attention to all elements of the route, not revealing what specific questions would be asked afterwards. The environment was created with Unity 3D software (version 2017.1.0f3), all models used as landmarks originated from the Unity asset store.

In line with the theoretical description in the introduction, five different tasks were designed to each represent functionally distinct domains of navigation ability. The video was followed by five tasks measuring landmark, location – egocentric, location – allocentric, path – route, and path – survey knowledge. As it was the only task including distractor items, the landmark task was always shown first, the order of the rest of the tasks was fully random. The landmark task entailed the presentation of eight items, four of which were present in the video, the other four were distractor items, leading to a chance level performance of 50%. Two different sets of landmarks were randomly assigned to participants, ensuring all 8 landmarks were used throughout all measurements. In the location – egocentric task, participants were shown a landmark and were asked which of six provided options showed an arrow pointing in the direction of the spaceship, at the end of the route. The six arrows would be exactly 60 degrees different from one another, covering 360 degrees in total. Chance level performance was therefore 16.7%, and a total of four trials were presented. Again, a random selection of landmarks was presented to each participant. It should be noted that this task design does not fully exclude the possibility of additional allocentric coding, as such configurational knowledge could support the formation of a directional response. However, this is very difficult to avoid for egocentric response to locations positioned further than one turn away from the viewpoint during task presentation, and responses still require taking a first person perspective within the route. For the location-allocentric task, participants were shown a landmark together with a map of the environment, with 4 possible locations indicated with the letters A, B, C, D. They were asked to indicate at which of the four locations the landmark was positioned. Therefore, chance level performance was at 25% for this task. Four trials were presented, one for each of four randomly selected landmarks. For one of the eight landmarks, the location in the response options was unintentionally presented with some deviation from the actual position. This trial was used in 5,921 out of 11,887 participants (including all adults). This error did not substantially affect performance on any of the other landmarks within this task (Bonferroni corrected alpha = 0.007), two-sided t-tests were all not significant, p > 0.03 in all comparisons) and had no significant effect on performance on the other tasks (p > 0.10 in all comparisons). Therefore, this trial was excluded and the mean performance of participants who received it was based on three instead of four trials. The path – route task entailed a response to the question in which direction the route continued for a given landmark. Depending on the landmark, two or three possible directions were provided; left, right, and straight ahead, mean chance level was 44% (range 37,5–50%). This was repeated for four randomly selected landmarks. The path – survey task consisted of 3 landmarks presented simultaneously, for which the two landmarks that were closest together should be selected. It was stressed that this should be measured from a bird’s eye perspective, and thus relying on the mental representation of the environment a participant had made. Specific attention was paid to stimulus selection for the sets of 3 stimuli, to ensure that responding to this task in terms of path order (stimulus has been seen nearby on while following the route) would lead to chance level performance. The task was repeated for four fixed sets of landmarks, presented in random order and positioning within each trial, with a chance level of 33.3%.

### Procedure and design

Participants started the experiment by providing informed consent by clicking the appropriate button on the opening screen, after reading the relevant information. For participants under the age of 16, explicit parental consent was requested. Next, the demographic and other descriptive questions were presented. This was followed by a screen indicating the video would be played when the participant clicked the ‘proceed’ button, warning them to be focused on the video and to avoid any distractions during the experiment. The video could not be paused. Next, the five tasks were presented and the participant could either finalize their participation or fill out a set of optional questions.

All participants received the same questions and tasks. The order of tasks was random, apart from landmark knowledge, which always came first, as the content of the other four tasks would improve landmark recognition. Every participant received a random selection of four landmarks for each task, to maintain a duration of 10 minutes for each participants, while allowing for a larger set of landmarks on the route.

### Statistical analyses - children

We studied navigation ability across the lifespan, by first focusing on development from childhood to young adulthood, followed by examining the aging process throughout adulthood. First, we verified if our tasks were suitable for all children in terms of difficulty, by calculating the proportion of children of each age to perform at or below chance level and the proportion of children with a perfect score, in comparison to young adults, aged 18–24. Next, we studied the developmental pattern for each task and examined the effects of age and gender on performance.

First, we assessed whether the difficulty level of the tasks was appropriate for this population. We did this by assessing how many children performed at or below chance level for each of the tasks, per age group. Additionally, we were also interested in how many children reached a perfect score, per age group and task. Frequency tables for each possible score were consulted to calculate the percentage of children with at or below chance level and perfect scores. As a reference, the young adults’ performance was included here.

Next, the effects of age and gender (male, female) were assessed for each task for children aged 8–17 and 18–24 year olds, by means of a MANOVA. Significant main and interaction effects of age were followed up by Bonferroni corrected post hoc analyses, in which alpha = 0.05/11 = 0.0045. Then, spatial anxiety and living area were added to this MANOVA and analysed for each of the tasks. Finally, the effect of spatial experience was analysed for each of the tasks with an ANOVA for all children within the 8–11 age range.

### Statistical analyses - adults

Participants were grouped based on age in the following increments: 18–29, 30–39, 40–49, 50–59, 60–69, 70–79, and 80–100. The effects of age and gender (male, female) were assessed for each task by means of a MANCOVA, with education level as a covariate. Significant main and interaction effects of age were followed up by Bonferroni corrected post hoc analyses, in which alpha = 0.05/7 = 0.0071. Next, spatial anxiety, living area, and spatial experience were added to this MANCOVA and analysed for each of the five tasks. Significant main effects of the additional variables were followed up by post hoc analyses.

## Results - Children

### Task difficulty

Table 3 shows the percentage of children scoring at or below chance level for each age group, for each of the tasks, as well as the percentage of children reaching a perfect score. For all five tasks a clear minority of children reach at or below chance level scores. Furthermore, perfect scores are found throughout all age groups and all tasks. Only the location – egocentric task shows that perfect scores are not present for all age groups, however, for this task, chance level is the lowest (16,7%) and the adult sample shows a 2% perfect performance.

**Table 3:** Performance of all children (age 8–17) on the navigation tasks, expressed by the percentage of participants performing at or below chance level and at perfect level. LM = landmark, LE = location egocentric, LA = location allocentric, PR = path route, PS = path survey.

Age group	% at or below chance level					% perfect score				
	LM	LE	LA	PR	PS	LM	LE	LA	PR	PS
8	2.7	11.0	23.3	15.1	30.1	34.2	0	11.0	20.5	15.1
9	5.8	18.6	32.6	17.4	25.6	39.5	1.2	7.0	14.0	9.3
10	5.1	24.2	26.3	18.2	23.2	42.4	0	13.1	15.2	11.1
11	3.6	21.4	23.8	17.9	29.8	41.7	0	8.3	21.4	9.5
12	2.2	19.6	19.6	16.3	14.1	39.1	1.1	14.1	12.0	12.0
13	3.6	19.6	35.7	10.7	19.6	41.1	0	12.5	21.4	8.9
14	7.1	25.7	24.8	8.0	23.0	48.7	2.7	16.8	23.0	9.7
15	4.7	9.4	23.4	7.8	17.2	45.3	0	26.6	37.5	10.9
16	3.8	18.5	20.7	8.7	28.3	35.9	1.6	20.1	31.0	9.8
17	2.9	18.4	23.0	10.9	19.0	44.8	0.6	18.4	27.0	9.2
18–24	2.2	20.3	16.5	8.3	21.3	46.0	2.0	19.6	29.0	10.7

### Age and gender

**Figure 2:**
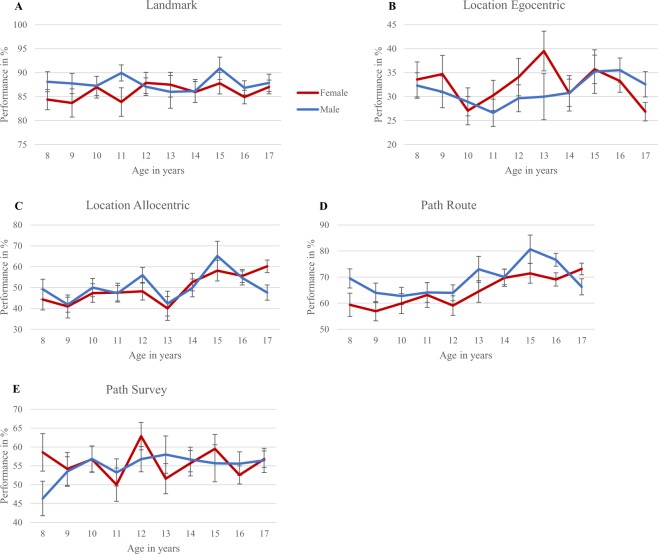
Mean performance of all children, for each age and gender on (**A**) Landmark task, (**B**) Location – Egocentric task, (**C**) Location – Allocentric task, (**D**) Path – Route task, (**E**) Path – Survey task. Error bars represent standard error of the mean.

The effects of age and gender were examined with a MANOVA. Nonbinary gender responses were possible for the adult participants and were excluded in this analysis, due to the inability to classify these responses in a meaningful way and their low occurrence (4 cases were found in the 18–24 year old group). Figure 2 depicts the mean performance per age group and gender, per task. For the landmark task the main effect of gender was at trend level, F(1,1002) = 3.16, p = 0.076, partial eta squared = 0.003, with males marginally outperforming females. For location – allocentric a significant main effect of age was found, F(9,1002) = 3.28, p < 0.01, partial eta squared = 0.029. The younger participants performed worse than the older participants, with the 9 year olds differing from the 15 year olds at trend level, p = 0.005, and significantly from the 18–24 year olds, p < 0.001. In addition, there was a similar difference between 13 and 18–24 year olds, p < 0.001. Visual inspection of the data substantiates this by showing a sudden increase in performance after 13 years of age. For path – route a significant main effect was found for age, F(9,1002) = 4.42, p < 0.001, partial eta squared = 0.038, and gender, F(1,1009) = 7.23, p < 0.01, partial eta squared = 0.007. Again, performance increased with age, in particular the 9 and 10 year olds significantly differed from the 18–24 year olds, p = 0.002 and p = 0.001, respectively, and males outperformed females. Visual inspection of the data supports this with a gradual increase in performance with age. All other main and interaction effects did not reach significance.

### Spatial anxiety, living area, and spatial experience

The factors spatial anxiety and living area (urban versus rural) were added to the MANOVA described above. This leads to the additional overall trend of living area, F(5,798) = 2.09, p = 0.065, partial eta squared = 0.013, with rural living area leading to marginally better performance than urban living area. For the landmark task, F(1,802) = 7.06, p < 0.01, partial eta squared = 0.009, and the path – survey task, F(1,802) = 3.97, p < 0.05, partial eta squared = 0.005, this effect was significant, in the same direction. Spatial anxiety did not significantly affect performance. The ANOVA including spatial experience did not reveal any significant effects of spatial experience.

## Results – Adults

As education level varied across age groups for the adults, this variable was added as a covariate to the analyses. As the number of participants reporting a non-binary gender was very small, these were not considered in analyses including gender.

### Age and gender

**Figure 3:**
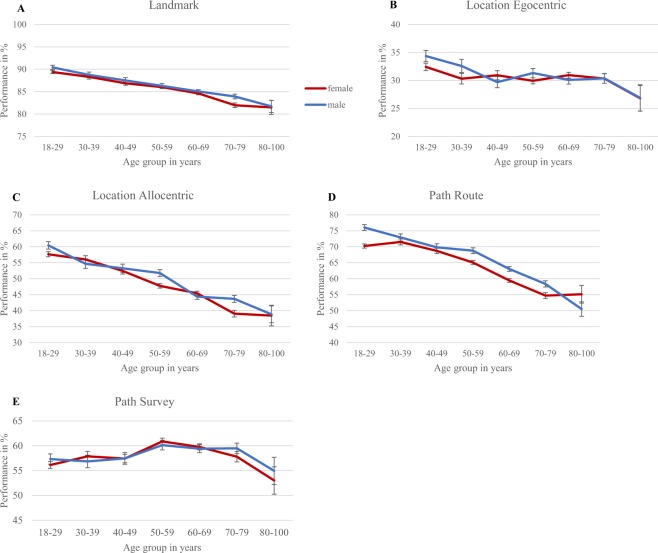
Estimated means of the performance of all adults, for each age group and gender on (**A**) Landmark task, (**B**) Location – Egocentric task, (**C**) Location – Allocentric task, (**D**) Path – Route task, (**E**) Path – Survey task. Error bars represent standard error of the mean.

The performance of all age groups and both genders for each of the five navigation tasks is depicted in Fig. 3. The MANCOVA with age group and gender as within subject factors and education level as a covariate revealed an overall main effect of both age group, F(30,43334) = 36.40, p < 0.001, partial eta squared = 0.020, and gender, F(5,10833) = 2.86, p < 0.05, partial eta squared = 0.001. Furthermore, the interaction of age group and gender was significant, F(30,43334) = 1.47, p < 0.05, partial eta squared = 0.001. Overall performance decreased with age group, and males outperformed females. The covariate education level also showed a significant main effect, F(5,10833) = 15.94, p < 0.001, partial eta squared = 0.007. As differential patterns of age group and gender were expected for each of the tasks, these significant effects were further examined for each task separately.

For the landmark task, there was a significant main effect of age group, F(6,10837) = 44.03, p < 0.001, partial eta squared = 0.024, and a trend level effect of gender, F(1,10837) = 3.67, p = 0.055, partial eta squared < 0.001. Performance gradually decreased with older age with performance being highest for the youngest adults (18–29 years) and worst for the two oldest age groups (70–100 years). Males performed slightly better than females. For the location – egocentric task only the main effect of age group was significant, F(6,10837) = 4.87, p < 0.001, partial eta squared = 0.003. Here the youngest age group (18–29 years) outperformed the older age groups (40–100 years) and no further decrease with age was present. For location – allocentric, the main effect of age group was also significant, F(6,10837) = 63,77, p < 0.001, partial eta squared = 0.034. A slight trend level effect of gender was found, F(1,10837) = 3.09, p = 0.079, partial eta squared < 0.001. The interaction of age group and gender also reached significance, F(6, 10837) = 2.87, p < 0.01, partial eta squared = 0.002. Performance linearly decreased from younger to older age groups with 18–29 year olds performing the best and 70–100 year olds performing the worst and males significantly outperformed females only for age groups 50–59 and 70–79. For the path – route task, again there was a significant main effect of age group, F(6,10837) = 93.54, p < 0.001, partial eta squared = 0.049. A significant main effect of gender, F(1,10837) = 9.64, p < 0.01, partial eta squared = 0.001 and interaction effect of age group and gender were also found, F(6,10837) = 2.38, p < 0.05, partial eta squared = 0.001. Performance linearly decreased with age, with highest performance for 18–29 year olds and lowest for 70–100 year olds. Males outperformed females for ages 18–29 and 50–79, but not for the other age groups. Furthermore, males showed a stronger aging effect than females, as the youngest males performed best and oldest males performed worst. Lastly, for the path – survey task, only a significant main effect of age was found, F(6,10837) = 6.12, p < 0.001, partial eta squared = 0.003. Here, the 50–69 year olds performed better than the youngest age group, no further age group differences were found. All other effects did not reach significance.

### Additional variables

To assess the impact of spatial anxiety, living environment and spatial experience, these three variables were added to the MANCOVA above. Overall, spatial anxiety showed a main effect at trend level, F(30,41006) = 1.41, p = 0.069, partial eta squared = 0.001. Spatial experience showed a significant main effect, F(15,2898.9) = 2.27, p < 0.01, partial eta squared = 0.001. Living environment did not reach significance. All three variables were examined for all five tasks separately. Spatial experience reached significance for the landmark task, F(3,10255) = 2.63, p < 0.05, partial eta squared = 0.001, and the location – allocentric task, F(3,10255) = 5.05, p < 0.01, partial eta squared = 0.001. In both cases lowest spatial experience showed worst performance (p < 0.05). Spatial anxiety was significant only for the landmark task, F(6,10255) = 2.16, p < 0.05, partial eta squared = 0.001. Here performance gradually decreased with higher levels of anxiety, with a significant difference between response 2 and 7 (p < 0.05).

## Discussion

We designed a novel task battery to assess navigation ability comprised of five tasks aimed at landmark, location, and path knowledge. The main aim was to create a detailed, large-scale depiction of navigation ability across the lifespan with appropriate measures. In addition, potential causes for the large individual variation in navigation ability were considered, by including gender, spatial experience, and spatial anxiety measures.

Across all ages, the task battery showed to be of an appropriate difficulty level, with no floor or ceiling effects for any of the participant groups, demonstrating it can be used for ages 8 and higher. In both the children and the adults clearly dissociable performance patterns were found for each of the tasks used. The *landmark knowledge task* tested the ability to remember landmarks viewed in the environment. This ability has already reached young adult level from 8 years of age and shows a shallow linear decline across adulthood. Performance for the elderly was lower, but still at a high level in an absolute sense (>80%). Whereas for children no gender effect was found, a male advantage was present for the adult sample, regardless of age group. Landmark knowledge is often not included when effects of age or gender are assessed, but the aging pattern found matches our prediction based on the suggested neural correlates. The age related changes in the parahippocampal place area and striatum^24,25^ agree with our finding of a the shallow decline with a relatively early onset. The male advantage for landmark knowledge agrees with reports on a male advantage in visual working memory (e.g.^41^). The *egocentric location task* assessed the ability to point to a specific location on the route, which showed no changes during childhood and highest performance for the youngest adults (18–29 years), and no effect of gender. This aging pattern is also in line with our predictions, based on the involvement of the parietal cortex, which is relatively stable in the aging process^8,27^. We proposed the involvement of geometric processing would favor males in task, which was not found. It could be that the egocentric perspective allowed females to use their preferred strategy and perform equally well^33^. It should be noted here that this task was relatively difficult. Furthermore, the possibility of allocentric processing in addition to the targeted egocentric coding cannot be fully excluded. Instead of indicating direction to a specific location from one’s current location, the type of response provided here does not exclude the more complex approach of additionally consulting an allocentric representation of the environment from which to derive directional information. In such a case a participant could switch from an egocentric to an allocentric perspective and back again to determine the correct answer. This possibility may contribute to the task difficulty observed here. Locations were indicated on a map in the *allocentric location task*, for which there was a clear increase across childhood, followed by a substantial linear decline throughout adulthood. A male advantage was found for two of the oldest age groups. The age effects found match our predictions, based on the later development of this complex cognitive process and its link to the hippocampus, particularly sensitive to aging (e.g.^26^). The male advantage can be linked to the explicit geometric nature of the task. In the *path route task*, participants were tested on their ability to connect landmarks to the respective turns that were taken along the route. This ability develops across childhood along with a male advantage, followed by a substantial linear decline in adulthood, which appears to be stronger in males. These findings match our hypothesis of early decline motivated by the involvement of the medial temporal lobe including the hippocampus in route retrieval^28^. Furthermore, there is a notable increase in performance from the age of 12 onwards, as predicted. Contrary to our prediction, the reliance on landmarks in this task does not affect females to the same extent as males. The quality of the mental representation of the environment was tested with the *path survey task*, in which distances between landmarks were to be compared. This was the only task for which a peak in performance was found late in adulthood, with no gender effects. We hypothesized an aging pattern, moderated by the involvement of brain regions less subject to aging effects, like the parietal cortex^29^. The aging pattern found shows that the moderation is substantial, even leading to a very late peak in this ability. The predicted gender effect for this task was absent; perhaps the strong reliance on landmark specific information could have eliminated a difference in performance between males and females.

Combined, these findings highlight the need for clear functional selection of navigation tasks matching the research questions at hand, as although tasks may seem highly related and all strongly linked to navigation behavior, performance can vary greatly across specific tasks. Moreover, when studying navigation ability as a whole, the current findings underline the importance of including each of these tasks, as they provide distinct input on an individual’s navigation ability. These findings can also be used to clarify contradictory effects of gender in existing literature. The gender effects found here strongly depend on both task type and participant’s age, which can vary substantially in gender comparison studies within the field of navigation (e.g.^32^).

In addition to age and gender, we also explored the potential contribution of other factors. Living environment, defined as rural or urban, has an impact limited to childhood and the landmark and path survey tasks. A possible interpretation of this effect is that living in an rural area, both the visual features of landmarks and the absolute distances between them are memorized better, due to a higher degree of visual access to landmarks and their spatial locations, as less visual occlusion is present in daily encounters with the immediate environment of children growing up in a rural area. The absence of impact of living environment in adulthood can possibly be explained by a variation in living environments across life, as only current living environment was asked for, not a history of changes in living environment throughout life. The amount of spatial experience, expressed by how frequently new locations are visited increased performance on landmark recognition, and the ability to point out locations on a map, indicating that spatial experience might boost explorative behaviour in adulthood. It appears that an increased exposure to novel environments stimulates our ability to memorize our surroundings and the ability to use maps, and apply this in a navigation context. In contrast to earlier reports (e.g. Walkowiak, Foulsham & Eardley, 2015), spatial anxiety has very limited impact on navigation ability and only showed to decrease performance on the landmark task, but none of the other domains. A potential hypothesis worth exploring in future work is that landmark recognition is highly realistic and may therefore trigger an emotional response such as anxiety more directly than the other domains. The operationalization of spatial anxiety should also be considered here. The concept of spatial anxiety has yet been ill-defined empirically, and it remains to be determined whether it is a specific, separate form of anxiety or a specific expression of general anxiety.

Strengths of the current study include the feasibility of the task battery for all included ages and the size and diversity of the sample of participants. With regard to the performance of children, the main aim was to examine the developmental patterns for each of the tasks in the online experiment. A sufficient amount of participants for each age group was found, with an acceptable gender distribution. The first examination of the data indicates that the difficulty level of the tasks used is appropriate for all young participants; only a small proportion of children perform at or below chance level and for four out of five tasks, optimal performance is also achieved in all age groups. The performance of adults allowed an examination of changes in navigation ability from young into old adulthood, which in combination with the developmental data provides a depiction of navigation ability across the lifespan. As with development, the results indicate differential aging patterns for each of the five tasks, and performance was sufficiently above chance level and below perfect performance for all age groups. In total, 11,887 participants took part in the experiment. This sample of participants showed a sufficient distribution across the different age groups and gender. Only the 80–100 age group was substantially smaller and skewed towards the lower end, in comparison to the other age groups. By requesting education level and the education system used in the Netherlands, which explicitly differentiates between students from the age of 12 onwards, based on intellectual capabilities, we were able to control for the variation in such cognitive factors. As education level varied between the different age groups in the adults, it was used as a covariate throughout the analyses in adults. In many of the existing aging studies on navigation, matching between older and younger adults is highly limited. Here we were able to adequately control for those factors and to report performance for a much more representative sample of the general population. This also provides a useful addition to earlier studies^4^, in which a smartphone app ‘Sea hero quest’ has been used to study navigation ability in a very large and heterogeneous sample of participants, to allow for detailed individual differences analyses. In comparison, the Sea hero quest data provides many datapoints originating from a wide range of nations based on multiple gameplays of navigation exercises. Apart from gender and age analyses, this has lead to an elaborate cross-cultural comparison of spatial abilities. Moreover, in this study effects of gender appeared to be related to a culture gender gap. The absence of strong gender effects in the current study could therefore be related to a lack of range in nationality, and the relatively low gender gap in the Netherlands. The potential impact of nationality and cultural factors deserves more attention in future studies. In contrast, with our current dataset we provide additional detail to existing literature by more specific differentiation within the functionally distinct aspects of navigation. Whereas the sea hero quest task design^4^ specifically addressed the ability to find a location after reading a map, and pointing towards one’s starting point, we attempted to create a more comprehensive measure of navigation ability by including complementary tasks covering the wide range of navigation ability. By individually assessing the domains of navigation, as motivated by patterns of navigation impairment, our current task design can be used in a clinical context in terms of diagnostic assessment.

It should be taken into consideration that the experiment was administered online, without direct supervision of an experimenter. This could increase the possibility of suboptimal performance. However, such effects are not expected to target specific subgroups of the sample tested, so should not affect the performance patterns in a relative sense. Also, in return for a potential source of noise in the data, a much larger sample of participants could be achieved. In collaboration with the Dutch government, the formal experimental set-up of the test was stressed in all communications to the general public, to minimize non-serious responses. In terms of future applications of the navigation task battery, continued online use of the task battery could be very helpful in settings in which for instance clinicians would like to screen for navigation impairment in large samples of participants. The current dataset would provide useful control dataset for those purposes, as it was created in highly similar circumstances. It should also be noted that apart from the individual factors we have included here, other factors could also make a significant contribution to navigation performance, such as small scale spatial abilities^42^ and personality traits^43^.

In short, the current study for the first time provides an in depth examination of navigation ability across dissociable functional domains. The outcomes highlight the impact of the specific navigation aspects assessed in determining effects of age and gender, which is highly important in interpreting current discrepancies in previous findings. For landmark knowledge a shallow linear decline with age was present, with a male advantage. Egocentric location knowledge shows an early decline with age, in absence of a gender effect. Allocentric location knowledge declines linearly with age, with a limited male advantage with older age. A substantial linear decline with age is found for path route knowledge, with a steeper decline in males. Path survey knowledge was markedly different with a peak in performance in late adulthood, in the absence of gender effects. The novel task battery presented here may be very useful as a brief, yet comprehensive tool to assess navigation ability in minutes and to detect potential navigation impairment in clinical populations and experimental settings.

## References

[1] Wolbers T, Hegarty M (2010). What determines our navigational abilities?. Trends Cogn. Sci..

[2] Wiener JM, Büchner SJ, Hölscher C (2009). Taxonomy of human wayfinding tasks: A knowledge-based approach. Spat. Cogn. Comput..

[3] Chrastil ER (2013). Neural evidence supports a novel framework for spatial navigation. Psychon. Bull. Rev..

[4] Coutrot A (2018). Global determinants of navigation ability. Curr. Biol..

[5] Barnes CA (1979). Memory deficits associated with senescence: A neurophysiological and behavioral study in the rat. J. Comp. Physiol. Psychol..

[6] Lester AW, Moffat SD, Wiener JM, Barnes CA, Wolbers T (2017). The aging navigational system. Neuron.

[7] Klencklen G, Després O, Dufour A (2012). What do we know about aging and spatial cognition? Reviews and perspectives. Ageing Res. Rev..

[8] Lithfous S, Dufour A, Després O (2013). Spatial navigation in normal aging and the prodromal stage of Alzheimer’s disease: Insights from imaging and behavioral studies. Ageing Res. Rev..

[9] Driscoll I (2013). The aging hippocampus: Cognitive, biochemical and structural findings. Cereb. Cortex..

[10] Simic G, Kostovic I, Winblad B, Bogdanovic N (1998). Volume and number of neurons of the human hippocampal formation in normal aging and Alzheimer’s disease. J. Comp. Neurol..

[11] Aguirre GK, D’Esposito M (1999). Topographical disorientation: a synthesis and taxonomy. Brain..

[12] van der Ham IJM, Martens MAG, Claessen MHG, van den Berg E (2017). Landmark agnosia: Evaluating the definition of landmark-based navigation impairment. Arch. Clin. Neuropsychol..

[13] Claessen MHG (2017). A systematic investigation of navigation impairment in chronic stroke patients: Evidence for three distinct types. Neuropsychologia..

[14] Blajenkova O, Motes MA, Kozhevnikov M (2005). Individual differences in the representations of novel environments. J. Environ. Psychol..

[15] Zhong JY, Kozhevnikov M (2016). Relating allocentric and egocentric survey-based representations to the self-reported use of a navigation strategy of egocentric spatial updating. J. Environ. Psychol..

[16] Claessen MHG, van der Ham IJM (2017). Classification of navigation impairment: A systematic review of neuropsychological case studies. Neurosci. Biobehav. Rev..

[17] Siegel AW, White SH (1975). The development of spatial representations of large-scale environments. Adv. Child. Dev. Behav..

[18] Ishikawa T, Montello DR (2006). Spatial knowledge acquisition from direct experience in the environment: individual differences in the development of metric knowledge and the integration of separately learned places. Cogn. Psychol..

[19] Kozhevnikov M, Motes MA, Rasch B, Blajenkova O (2006). Perspective-taking vs. mental rotation transformations and how they predict spatial navigation performance. Appl. Cognitive Psychol..

[20] Nardini M, Burgess N, Breckenridge K, Atkinson J (2006). Differential developmental trajectories for egocentric, environmental and intrinsic frames of rerefence in spatial memory. Cognition..

[21] Janzen G, Jansen C, van Turennout M (2008). Memory consolidation of landmarks in good navigators. Hippocampus.

[22] Klatzky, R. L. Allocentric and egocentric spatial representations: definitions, distinctions, and interconnections. In Freksa, C., Habel, C. (Eds.) Spatial Cognition. An Interdisciplinary Approach to Representing and Processing Spatial Knowledge, pp. 1–17, Springer (1998).

[23] Montello, D. R. A new framework for understanding the acquisition of spatial knowledge in largescale environments. In Egenhofer, M. J. & Golledge, R. G. (Eds.), Spatial and temporal reasoning in geographic information systems (pp. 143–154). New York: Oxford University Press (1998).

[24] Epstein RA (2008). Parahippocampal and retrosplenial contributions to human spatial navigation. Trends Cognit. Sci..

[25] Chan E, Baumann O, Bellgrove MA, Mattingley JB (2012). From objects to landmarks: the function of visual location information in spatial navigation. Front. Psychol..

[26] Burgess N, Maguire EA, O’Keefe J (2002). The Human Hippocampus and Spatial and Episodic Memory. Neuron.

[27] Colombo D (2017). Egocentric and allocentric spatial reference frames in aging: A systematic review. Neurosci. Biobehav. Rev..

[28] Brown TI, Hasselmo ME, Stern CE (2014). A high-resolution study of hippocampal and medial temporal lobe correlates of spatial context and prospective overlapping route memory. Hippocampus.

[29] Shelton AL, Gabrieli JDE (2002). Neural correlates of encoding space from route and survey perspectives. J. Neurosci..

[30] Cohen R, Schuepfer T (1980). The representation of landmarks and routes. Child Dev..

[31] Pine DS (2002). Neurodevelopmental aspects of spatial navigation: A virtual reality fMRI study. NeuroImage.

[32] Coluccia E, Louse G (2004). Gender differences in spatial orientation: A review. J. Environ. Psychol..

[33] Grön G, Wunderlich AP, Spitzer M, Tomczak R, Riepe MW (2000). Brain activation during human navigation: gender-different neural networks as substrate of performance. Nat. Neurosci..

[34] Sandstrom NJ, Kaufman J, Huettel SA (1998). Males and females use different distal cues in a virtual environment navigation task. Cogn Brain Res.

[35] Maguire EA (2000). Navigation-related structural change in the hippocampi of taxi drivers. Proc. Natl. Acad. of Sci..

[36] Maguire EA, Woollett K, Spiers HJ (2006). London taxi drivers and bus drivers: A structural MRI and neuropsychological analysis. Hippocampus.

[37] Walkowiak S, Foulsham T, Eardley AF (2015). Individual differences and personality correlates of navigational performance in the virtual route learning task. Comput. Human Behav..

[38] Verhage, F. Intelligentie en leeftijd: Onderzoek bij Nederlanders van twaalf tot zevenenzeventig jaar. Assen: Van Gorcum (1964).

[39] Claessen MHG, Visser-Meily JMA, de Rooij NK, Postma A, van der Ham IJM (2016). The Wayfinding Questionnaire as a self-report screening instrument for navigation-related complaints after stroke: Internal validity in healthy respondents and chronic mild stroke patients. Arch. Clin. Neuropsychol..

[40] De Rooij NK, Claessen MHG, van der Ham IJM, Post MWM, Visser-Meily JMA (2017). The Wayfinding Questionnaire: A clinically useful self-report instrument to identify navigation complaints in stroke patients. Neuropsychol. Rehab..

[41] Pauls F, Petermann F, Lepach AC (2013). Gender differences in episodic memory and visual working memory including the effects of age. Memory.

[42] Hegarty M, Montello DR, Richardson AE, Ishikawa T, Lovelace K (2006). Spatial abilities at different scales: Individual differences in aptitude-test performance and spatial-layout learning. Intelligence.

[43] Burles F (2014). Neuroticism and self-evaluation measures are related to the ability to form cognitive maps critical for spatial orientation. Behavioural Brain Research.

